# Tissue Equivalents Based on Cell-Seeded Biodegradable Microfluidic Constructs

**DOI:** 10.3390/ma3031833

**Published:** 2010-03-15

**Authors:** Jeffrey T. Borenstein, Katie Megley, Kimberly Wall, Eleanor M. Pritchard, David Truong, David L. Kaplan, Sarah L. Tao, Ira M. Herman

**Affiliations:** 1 Biomedical Engineering Center, Draper Laboratory, 555 Technology Square, Cambridge MA, USA; E-Mails: kmegley@berkeley.edu (K.M.); kwall@draper.com (K.W.); stao@draper.com (S.L.T.); 2 Department of Biomedical Engineering, Tufts University, 4 Colby Street, Medford MA, USA; E-Mails: eleanor.pritchard@tufts.edu (E.M.P.); david.kaplan@tufts.edu (D.L.K.); 3 Center for Innovations in Wound Healing Research, Tufts University School of Medicine, 150 Harrison Avenue, Boston MA, USA; E-Mail: dtruong@tufts.edu (D.T.)

**Keywords:** microfluidics, microfabrication, tissue engineering, wound healing, vasculature

## Abstract

One of the principal challenges in the field of tissue engineering and regenerative medicine is the formation of functional microvascular networks capable of sustaining tissue constructs. Complex tissues and vital organs require a means to support oxygen and nutrient transport during the development of constructs both prior to and after host integration, and current approaches have not demonstrated robust solutions to this challenge. Here, we present a technology platform encompassing the design, construction, cell seeding and functional evaluation of tissue equivalents for wound healing and other clinical applications. These tissue equivalents are comprised of biodegradable microfluidic scaffolds lined with microvascular cells and designed to replicate microenvironmental cues necessary to generate and sustain cell populations to replace dermal and/or epidermal tissues lost due to trauma or disease. Initial results demonstrate that these biodegradable microfluidic devices promote cell adherence and support basic cell functions. These systems represent a promising pathway towards highly integrated three-dimensional engineered tissue constructs for a wide range of clinical applications.

## 1. Introduction

Recently, significant progress has been made in the development of engineered tissues for clinical applications including wound and burn repair. However, progress towards engineering complex tissues and organs has been limited by challenges in forming an integrated and functional vasculature. In highly metabolic organs such as the heart, lung and liver, capillaries must reach within 100–200 μm of each cell within the growing tissue to provide adequate gas, nutrient and metabolite exchange [1]. Insufficient vascularization can lead to improper cell integration or cell death in engineered tissue constructs. While endogenous blood vessels can invade the implanted tissue enabling spontaneous blood vessel formation, the rate of neovascularization for millimeter-sized implants typically takes several weeks, such that clinically useful implants would not re-vascularize in a clinically-relevant timeframe [2]. Thus, achieving a replacement tissue with a patent and sustainable microvasculature represents a key, rate limiting step in the formation of most replacement tissues or organ systems.

Methods for vascularization that would enable generation of highly metabolic replacement tissues comprise various pathways. One approach utilizes functionalized scaffold materials to induce or sustain the host’s angiogenic activity *in vivo*. Here, highly permeable hydrogels or porous materials have been utilized as scaffold matrices, not only to increase bulk diffusion of oxygen and nutrients, but also to encourage vessel infiltration [3,4]. Both the mechanical stability and the high degree of pore interconnectivity required represent challenges for these approaches. In addition to scaffold mechanical and structural properties, this approach elicits localized angiogenesis through the use of bio-active scaffolds which have been chemically derivatized by incorporating angiogenic inducers, *i.e.*, small molecules, growth factors or extracellular matrix components that can be released locally and stimulate angiogenesis *in situ* [5]. Use of chemical signals to promote vascularization has resulted in the successful formation of capillary beds in both synthetic and naturally-occurring materials [6]. However, robustness and stability of nascent capillary beds generated using these growth-factor-based approaches remain a concern.

A second general approach toward achieving tissue vascularization involves precisely engineering three dimensional matrices with microfluidic conduits that mimic the tissue-specific features of the endogenous microcirculation. In this case the scaffold material provides the physical template needed to organize cells into a functional microvasculature, achieving patency and enabling engineered tissue- or organ sustainability. In this regard, the microfluidic network enables mass transfer of nutrients in exchange for metabolites and waste from the scaffold. Furthermore, mechanical forces and cues such as fluid shear can be designed within the microfluidic network to insure healthy tissue- or organ-specific physiology. Three dimensional tissue scaffolds containing an engineered microvasculature have been fabricated from a number of biocompatible polymers. The capabilities of soft lithography and polymer-based BioMicroElectroMechanical systems (BioMEMS) have provided tools for the fabrication of biocompatible polymer systems. Previously reported materials used for three dimensional scaffold molding include polydimethylsiloxane (PDMS) [7], polycaprolactone (PCL) [8], poly(lactic-co-glycolic acid) (PLGA) [9,10], and polyglycerol sebacate (PGS) [11,12,13]. Other approaches [14] have demonstrated vascularization of tissues such as skeletal muscle tissue by seeding endothelial cells in a vascularized construct, which, after implantation, led to attraction of host blood vessels towards the engineered construct.

For *in vivo* applications, there are several critical scaffold properties required in order to properly support microvascular development. Mechanically, the material must have high tensile strength while providing a flexible and elastomeric structure [15]. It is also important that fabrication of the scaffold should involve mild processing conditions that can be carried out relatively simply, reliably, and inexpensively. To perform *in vivo*, microvascular scaffolds must be biocompatible, non-thrombogenic, and resistant to infection [16]. In addition, scaffolds must promote cellular adhesion, cell growth, and retention of differentiated cell types or contain chemical moieties for modification to do such. Finally, the material should also be designed to degrade at a rate that is compatible with the time required to achieve mechanical integrity while enabling microvascular stabilization and patency.

Here, we investigate silk fibroin as a suitable biomaterial for the construction of a biodegradable microvascular scaffold. Purified from the domesticated silk worm *Bombyx mori,* silk fibroin has been extensively characterized and proven effective in clinical applications [17] as early as forty years ago for surgical sutures [18]. Silk fibroin continues to be the subject of intensive investigation for numerous biomedical applications [19,20,21,22]. An important recent development was the discovery that sericin (the glue like protein which accounts for 20–25% by weight of the silk produced by silkworms and can be biochemically separated from the silk fibroin, itself) was responsible for the inflammatory responses observed in native silk. By extracting the residual sericin from silk fibroin, a suitable non-immunogenic biomaterial can be formed. In addition to its low immunogenicity, silk fibroin purified from *B. mori* cocoons demonstrates superior mechanical strength and elastic properties compared with virtually all other biopolymers [23]. Further, the availability of amine and acid side chains on the silk fibroin protein, itself, make chemical or covalent modification with a vast number of conjugating or functionalizing reagents possible. This allows for a tailoring of the silk material that might foster site- and or cell-specific microdomains offering optimized scaffold environments required for normal cell growth, differentiation and tissue morphogenesis. Silk fibroin is also biodegradable via proteolysis; and, the degradation rate can be precisely tuned. For example, during the first five day period of enzyme exposure, a pure silk fibroin film loses roughly 10% of its original mass, followed by an extended period of slow degradation lasting 1 to 2 years [24]. Slow degradation *in vivo* is ideal as it allows for the gradual incorporation of native cells as the implanted structure is slowly removed [17]. Silk is also an FDA approved biomaterial. The dense, nonporous nature of the silk scaffold we are reporting serves only as an initial demonstration vehicle for this technology, and therefore the porosity and structural properties of future silk scaffolds may also be tuned to provide more ideal transport properties and cell-cell communication in the engineered tissue constructs. Thus, the inherent properties and tenability of silk fibroin make it an ideal candidate for implantation as a microvascularizing scaffold biomaterial. Building on the initial demonstration of a silk-based microfluidic system for tissue constructs [15], this is the first report of a non-inflammatory, non-immunogenic high-strength silk fibroin based microvascular scaffold.

## 2. Results and Discussion

### 2.1. Microfabrication of Tissue Scaffold

Silk microfluidic scaffolds were fabricated using techniques described in detail in the Methods section. Briefly, silicon wafers were patterned using a photolithographic process wherein a positive pattern of interconnected trenches represents the microchannel network. These structures were then used to produce PolyDiMethylSiloxane (PDMS) transfer molds, since de-molding of the silk films requires an elastomeric mold such as PDMS rather than the rigid silicon master mold. These PDMS transfer molds were then used to cast silk fibroin trenched layers, which were then bonded to flat silk films to form closed microchannel networks. Bonded devices were assembled with inlet and outlet tubing to enable flow of media and cellular inoculation.

**Figure 1:**
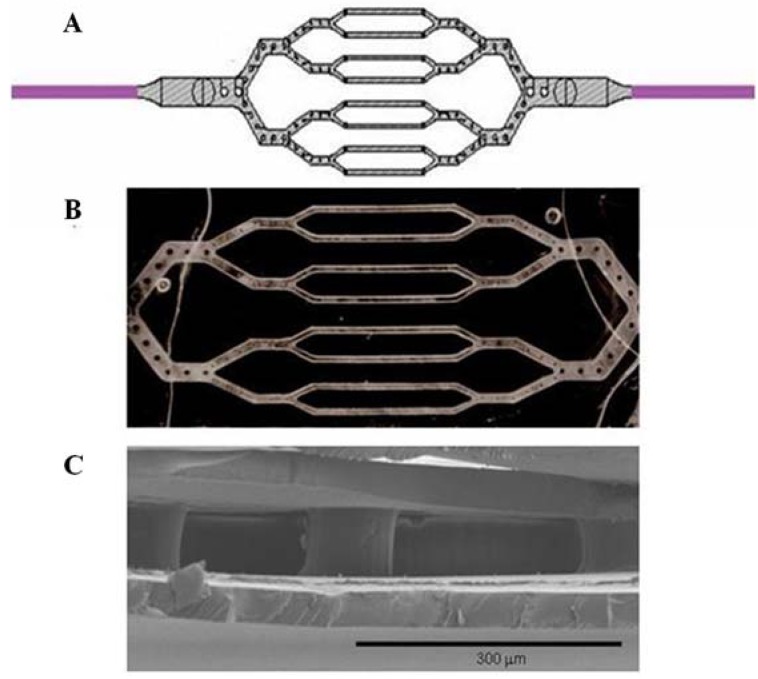
(A) L-Edit drawing of a network design layout containing round microposts. (B) Brightfield image (1x) of the patent micromolded, bonded silk network. Scale bar = 100 μm. (C) Scanning electron microscope image of the silk microfluidic channel and bioerodible microposts (white arrow).

Figure 1 illustrates the process of generating a design to create a branched, bifurcated microvascular network, much like that existing in within the human dermal microcirculation. The fabrication and production processes yield a layout with special design features that contain physiologically size-relevant microchannels and supporting posts (Figure 1; A), which are comparably fabricated from silk and provide structural support that fosters device patency and optimal flow characteristics for cellular seeding of the construct.

Silk microfluidic devices are able to support suitable flow rates without leakage and delamination, permitting fluid flow at rates compatible with cellular inoculation and device seeding. Human dermal microvascular endothelial cells (HDMVECs) were delivered into the device under flow, allowed to attach for an initial period, and following the attachment period, devices harboring living cells were perfused with fresh media at steady flow rates. Cell culture was continued over a period of roughly one and a half weeks. Importantly, however, we also observed that the yield of viable devices was adversely impacted by the presence of stress-induced cracks that sometimes emerged. Typically, the source of these stresses and methods were derived from rapid drying, variations in film thickness near the edge of the cast layers, as well as intrinsic mechanical stresses from the thermal excursions during the process. In certain cases, we reasoned that modifying agents able to be blended into the silk solution during processing might reduce these stresses [25]; such efforts to address the yield loss are currently being pursued. For devices without stress-induced cracks, flow and cell seeding proceeded smoothly and cellularization results are described in the next section.

### 2.2. Evaluation of Cell-Seeded Construct

**Figure 2:**
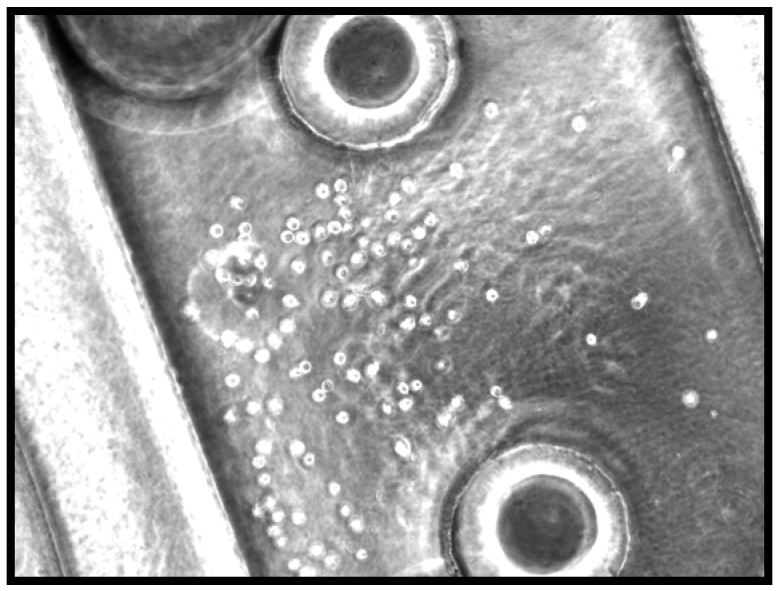
Microvascular Device Cell Seeding after 2 Days in Culture.

In order to verify the presence and condition of HDMVECs in the construct, live cell imaging was conducted on the device using phase contrast microscopy (Figure 2). Phase contrast imaging found many rounded, trypsinized cells within the microchannel network at each level of branching and bifurcation. As can be seen in Figure 2, there was a high density of rounded and refractile cells that settled and anchored in between posts of an intermediate channel immediately after seeding. Ultimately, these cells spread and proliferate and can be observed at consistent concentrations throughout the entire device.

**Figure 3:**
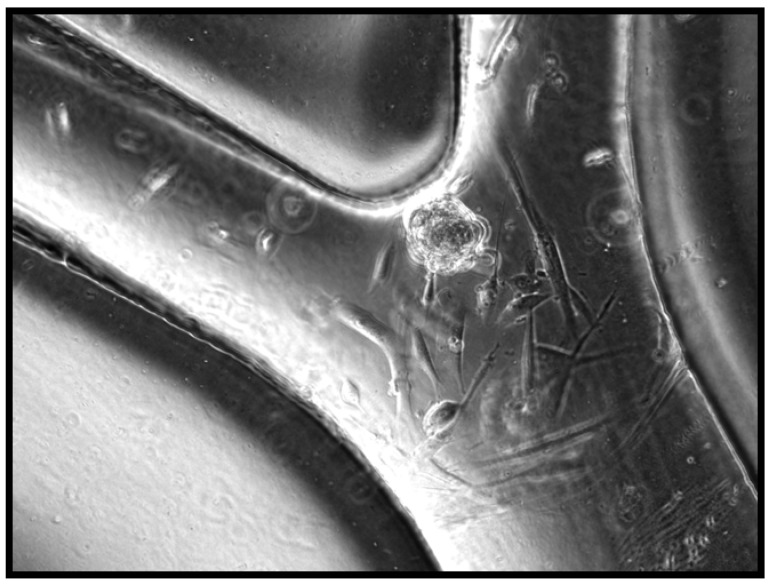
Seeded Device 2 Days after Media Perfusion.

After 2 days of media perfusion at 1 μL/min in the incubator, cells were attached and spreading as seen in Figure 2. A small cluster of unattached cells can be seen at the branch of a channel. This was more frequently observed at early time points, since unattached cells had not yet been flushed out of the system completely. At six days post perfusion, the cells remain attached to interior walls of device on two different planes, most likely the top and bottom of the channel. The flow of 1 μL/min of supplemented media keeps the cells alive and attached within the closed silk device. Attached cells were found to proliferate and elongate in a fashion characteristic of HDMVEC, as observed in Figure 3.

### 2.3. Staining of Flat Cell-Seeded Silk Films

**Figure 4:**
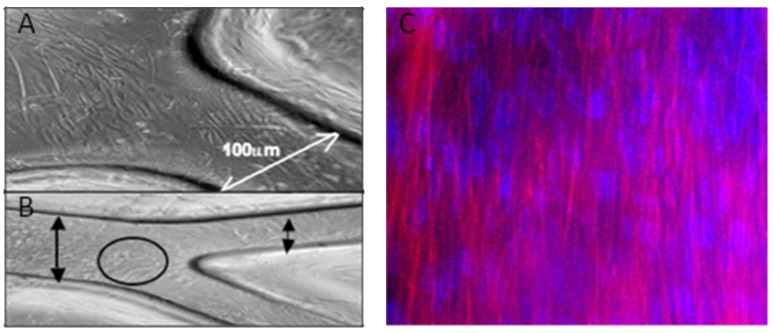
(A) Endothelial seeding after 3 days at site of bifurcation (closeup) and (B) Lower magnification view of (A). (C) Simultaneous fluorescence-based imaging of human dermal microvascular endothelial cells cultured on flat silk films (Red = Alexa 586- Phalloidin, F-Actin containing stress fibers are axially oriented; Blue = Hoechst Dye, Nuclei).

Fluorescent phalloidin, which binds filamentous (F-) actin, and Hoechst dye, which intercalates within the nuclear compartment, were used to examine cells seeded on silk films to cell shape, cytoskeletal and nuclear arrays. These stains could not be used inside closed, seeded microfluidic silk devices due to the inherent autofluorescence characteristic of silk, which was amplified 4—fold due to the walls of the microfluidic channel. For these purposes, HDMVECs were cultured on open channels or flat sheets of untreated silk fibroin for optimization of fluorescence-based imaging. Figure 4 shows a confluent, differentiated HDMVEC layer populating a silk film three days after seeding, in a static control device, in order to demonstrate the compatibility of the silk film with the formation of a confluent HDMVEC layer.

Silk fibroin microvascular scaffolds were seeded and maintained in perfusion culture for periods up to 9 days. During this time HDMVEC cells showed characteristic growth and alignment inside the channels of the device. The silk scaffold itself remained physically stable during this time and did not degrade substantially as to result in a loss of mechanical integrity.

### 2.4. Discussion

Silk fibroin is a candidate biomaterial for tissue engineering scaffolds based upon its unique mechanical properties, biodegradability and biocompatibility [17]. Along with these advantageous properties of silk, use of material that has received FDA approval for certain clinical applications for scaffold fabrication, provides an additional benefit regarding ease of use and acceptance within the medical field. Beyond the inherent properties and benefits of silk as a scaffolding material, this work has demonstrated that microfluidics-based construction of microvascular networks from lithographically fabricated master molds will enable the formation of an integrated microvasculature for tissue engineering applications. The process relies upon the use of PDMS inverse molds to replicate silk microchannel layers from high resolution silicon masters, resulting in highly reproducible devices and consistency between batches. Lastly, the entire device fabrication process is robust and tunable to the needs of specific systems.

In the present study, the compatibility of silk fibroin constructs and HDMVEC cells seeded in microchannel networks designed to mimic the bifurcating nature of vasculature has been demonstrated. There remains much work to be done to improve the simplicity and yield of functional devices, however, based on ongoing materials science and engineering studies with silk proteins these issues are fully addressable, including improved toughness, plasticization and new silk adhesive systems.

## 3. Experimental Section

### 3.1. Preparation of Silk Solution

Aqueous silk fibroin solutions were made using a modified extraction procedure of *B. mori* cocoons based on methods previously described [27]. Sectioned silk cocoons were boiled for 45 minutes in an aqueous solution of 0.2M Na_2_CO_3_ to extract sericin proteins. The purified silk fibroin was then washed thoroughly with de-ionized (DI) water and allowed to dry overnight at ambient conditions. The silk fibroin was then dissolved in aqueous 9.3 M LiBr at 60 °C for 4 hours. The resulting solution was dialyzed against DI water using a Slyde-a-lyzer cassette with a 3,500 MWCO for a period of 48 hours [26]. The resultant silk fibroin solution (aqueous 6–8% weight/weight; w/w) was further concentrated by a reverse dialysis method using 30 wt % poly(ethylene glycol) (PEG) [27,28]. The final working silk fibroin solution 10–13% (w/w) was then available for scaffold fabrication.

### 3.2. Silk Microvascular Network Scaffold Fabrication

**Figure 5:**
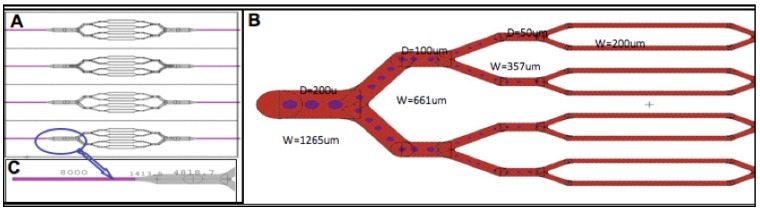
(A) Design of multiple bifurcated networks, (B) Detailed layout of network with supporting posts, (C) Magnified view of bifurcation.

The design of the microvascular channels were carefully chosen to mimic a terminally branched microvascular perfusion circuit observed within specific organ systems, e.g. human dermal microvasculature. However, both branched bifurcated terminal microvascular networks or collateralized networks can be fashioned so that the entrance channels measure 1265 µm in width, which can branch or bifurcate evenly. In the studies described, herein, entrance channels have been designed to bifurcate 3 times in series creating channels of width 600, 350 and 200 µm respectively. These channel widths were reproduced quite closely in the actual silk scaffold; contraction/expansion of the structure upon silk molding was limited to a few percent or less. Smaller channels were considered, but were reserved for future studies due to challenges associated with seeding microchannels with dimensions close to those of capillaries. In addition, the fabrication mask and design layout were engineered to include pillar-like posts; structures that are spaced throughout the microchannel pathways, aimed at ensuring channel patency during the layer bonding process. These design iterations included round and oval pillars, which also provide additional surface area for cell attachment as shown (Figure 5).

Channel layouts were created using microfabrication software (L-Edit, Tanner Research, Monrovia CA) and printed onto transparent flexible photomasks using a laser printer with 5080 dpi resolution (Pageworks). These photomasks were used to transfer microchannel network patterns into SU-8 2000 photoresist (Microchem, Newton MA) coated 100mm silicon wafers using a mask aligner (Karl Suss, Waterbury VT). The height of the SU-8 layer determines the depth of the microchannels, and is governed by the specific SU-8 product and the spin speed during SU-8 deposition on the wafers. Lithographic masters are postbaked and then surface-treated to enable mold release using a Teflon-like coating layer deposited in a reactive ion etcher (STS, Newport UK). The wafer patterns were then transferred to poly-dimethylsiloxane (PDMS) by casting a 10:1 mix of polymer:curing agent of Sylgard 184 (Dow Corning, Midland MI) onto the wafer and curing for 3 h at 65 °C. The PDMS inverse mold was then peeled off the silicon wafer.

For creating branched bifurcated microchannel networks in silk fibroin, purified and concentrated silk solution was cast over both the microchannel inverse PDMS mold as well as a flat section of PDMS and allowed to dry for 48 hours at ambient conditions. Flat sections were cut from the molded silk layers to avoid working with curved areas near the edge of the film. Silk films were removed from their PDMS mold and treated with 50% volume/volume(v/v) methanol in water for 4 hours to increase the beta sheet content and therefore the water stability [26] prior to a final DI water wash (1 hr at room temperature, RT). Microfluidic devices were then formed by joining microchannel and flat silk films, and two flexible tip needles (for entry and exit flow). The entire stack, which includes a bonding solution of aqueous 6 wt % silk deposited by spinning or dip-coating at the interface is then laminated under pressure at 70 °C for 18 hours [15]. When applied with the appropriate thickness, the bonding solution did not substantially affect the geometry of any of the microchannels in the network. The devices were removed from the bonding process and anchored between two glass microscope slides to create a controlled culture environment. UV sterilization can then be readily accomplished

### 3.3. Cell Culture

All cell culture reagents were purchased from GIBCO (Carlsbad, CA) unless otherwise noted. Well characterized human dermal microvascular endothelial cells (HDMVECs) were cultured with Dulbecco’s modified Eagles media (DMEM) supplemented with 5–10% fetal bovine serum, 1% penicillin streptomycin fungizone antimyotic (PSF), and 2.5 mM *4-(2-hydroxyethyl)-1-piperazineethanesulfonic acid* (HEPES) [29]. Experiments were performed using media containing 5–10% serum with no significant impact on endothelial proliferation. Passage 7–10 cells used for experiments were cultured at 37 °C and 5% CO­_2_ with fresh media supplied twice per week during growth. HDMVECs were allowed to remain one day post-confluent HDMVEC cultures were harvested by trypsinization (Trypsin/EDTA, GIBCO, Carlsbad, CA) as previously reported [28,29].

### 3.4. Cell Seeding

Immediately following trypsinization, the HDMVEC cell pellet was suspended to a concentration of 1 × 10^6^ cells/mL in growth media supplemented with 10ng/mL of basic fibroblast growth factor (bFGF) and platelet derived growth factor (PDGF-BB) to enhance cell proliferation after seeding [29]. Two hours prior to trypsinization and HDMVEC re-suspension, the channels of the silk microdevices were saturated with supplemented media and allowed to condition for 2 hours. The concentrated cell solution was then dropped into fluidic connections just before the entrance needle to the device. Behind the cell solution a media line was connected and pushed by syringe pump (Harvard Apparatus, Holliston MA) at a flow rate of 5 μL/min until cells were visible inside the device. Cells were allowed 16 hours of static attachment at 37 °C and 5% CO­_2_ before initiation of media perfusion (1 μL/min).

### 3.5. Cell Staining

Flat sections of silk, used as a static control in order to enable full visualization of the endothelial layer, with attached HDMVEC cells were fixed and permeabilized to facilitate cytoplasmic and nuclear localization of F-Actin (Alexa586- Phalloidin (Molecular Probes, OR) and DNA (Hoechst, Molecular Probes, OR) In this way, cellular colonization within the branched bifurcated microchannel networks could be evaluated and cellular morphology simultaneously assessed. Media was aspirated from the cell culture and films were washed three times with PBS prior to fixation at room temperature 3.7% v/v formaldehyde (Sigma-Aldrich, St. Louis, MO) for 10 minutes. The fixative solution was removed and the films were again washed three times with PBS prior to permeabilization with 0.1% v/v Triton in PBS for 5 minutes. Following permeabilization, cell containing films were again rinsed with PBS three times. To prevent non-specific staining, films were incubated with 1% BSA in PBS (Sigma-Aldrich, St. Louis, MO) solution for 20 minutes at room temperature. The BSA solution was aspirated and 0.165 µM phalloidin (Invitrogen Molecular Probes, Eugene, OR) was added and allowed to incubate for 20 minutes at room temperature while protected from light. The cell containing films were again washed with PBS three times. Next, Hoechst 33342 (Invitrogen Molecular Probes, Eugene, OR) at 2 µg/mL in PBS was added and allowed to incubate for 20 minutes at room temperature while also protected from light. Finally, the Hoechst solution was aspirated and the films were washed with PBS prior to light microscopic imaging.

## 4. Conclusions

This work has demonstrated that biodegradable microfluidics technology can be used to generate microvasculature for applications in tissue engineering and wound healing using a clinically useful biopolymer, silk fibroin. Microfabrication technology is capable of producing patent, leakproof microchannel networks, and the silk surface supports the formation of a confluent endothelial layer. Initial staining and cell viability assessment suggests that the endothelium is viable and suitable for establishment of an engineered microvasculature.

There are several potential directions for further expanding and extending this work. One potential avenue for this work would be to incorporate a co-culture system with organ-specific cell types on both the interior and exterior of the scaffold. For instance, seeding keratinocytes on the exterior of the scaffold would enable the development of vascularized skin replacement and wound repair constructs. To enable interaction between keratinocytes and vascular cells, a porous silk membrane could be inserted between the two compartments. This would enable skin cells to be placed proximal to the endothelialized channels, and these cultured skin cells would be fed by nutrients and oxygen through the vascular network. Once implanted, the endothelialized networks would provide be anastomosed to the host vasculature, providing an immediate vascular pathway for the engineered construct. Another possible extension of this work would be to multiplex individual microvascular networks to create a large-scale culture system. Previous efforts have been reported to both vertically stack and also linearly connect flow systems in other biomaterials such as PDMS [30]. This direction would be crucial in demonstrating the ability to create large-scale vascularized tissues in appropriate characteristic length scale in all three dimensions.
